# Case Report: Resetting the Humoral Immune Response by Targeting Plasma Cells With Daratumumab in Anti-Phospholipid Syndrome

**DOI:** 10.3389/fimmu.2021.667515

**Published:** 2021-04-12

**Authors:** Daniel E. Pleguezuelo, Raquel Díaz-Simón, Oscar Cabrera-Marante, Antonio Lalueza, Estela Paz-Artal, Carlos Lumbreras, Antonio Serrano Hernández

**Affiliations:** ^1^ Department of Immunology, Hospital Universitario 12 de Octubre, Madrid, Spain; ^2^ Department of Internal Medicine, Hospital Universitario 12 de Octubre, Madrid, Spain

**Keywords:** anti-phospholipid, refractory, treatment, daratumumab, anti-CD38

## Abstract

**Introduction:**

Monoclonal antibodies (mAb) targeting plasma cells are malignant gammopathy designed and approved therapies. In recent years, these antibodies have also been increasingly introduced for non-malignant conditions such as autoimmune-mediated diseases. The Anti-Phospholipid Syndrome (APS) is an immune-mediated disorder in which autoantibodies against phospholipid associated proteins could elicit the activation of the coagulation cascade in specific situations. Therefore, the mainstream treatment for APS patients is the use of anticoagulant therapy. However, there are refractory patients who would benefit from targeting the antibodies rather than their effects. Rituximab, a B-cell depleting mAb, and intravenous immunoglobulins (IVIG) have been used in APS patients without showing a clear beneficial effect or a significant drop in anti-phospholipid antibody (aPL) levels.

**Clinical case:**

We present our first APS case treated with daratumumab, an anti-CD38 mAb, in a 21-year-old patient with APS who presented with recurrent venous thromboembolic events despite adequate anticoagulant therapy. She tested positive for lupus anticoagulant, anti-cardiolipin IgG, anti-beta-2-glycoprotein-I IgG and anti-phosphatidylserine/prothrombin IgG and IgM. She was administered one dose weekly of daratumumab for 4 weeks. The treatment showed an adequate safety profile and was well tolerated. The patient was discharged after undergoing a clinically significant improvement. After the therapy, her levels of positive aPL declined significantly and most continued to decrease during the next three months. The patient experienced a new thrombotic episode two years after the therapy associated with poor adherence to antithrombotic therapy.

**Conclusions:**

The treatment with daratumumab showed an adequate safety profile, was well tolerated and led to a significant clinical improvement. Levels of aPL lowered on therapy and the next three months and then rose again during follow-up. Further investigation is needed to better elucidate the role and optimal timing and doses of daratumumab in treatment of refractory APS.

## Introduction

Antiphospholipid syndrome (APS) is an autoimmune disorder characterized by vascular thrombosis and/or pregnancy morbidity in the presence of persistent antiphospholipid antibodies (aPL) (1). Antibodies against beta-2-glycoprotein I (aB2GPI) and Cardiolipin (aCL), together with the functional assay lupus anticoagulant (LA), are the three laboratory tests considered for classification of this Syndrome in the revised Sydney criteria (1).

Since thrombosis is the most relevant clinical manifestation of this disorder, its treatment relies almost exclusively on anticoagulant therapy with warfarin, acenocoumarol and heparin-based regimens. EULAR recommendations from 2019 suggest assessing the anticoagulation range using International Normalized Ratio (INR)) (2). If the INR is found to be lower than the target, the patient would need the anticoagulant therapy to be adjusted to maintain the INR within the therapeutic ranges (INR: 2–3) (3). If the INR is within the therapeutic ranges at the time of a thrombotic episode, it is recommended to increase the intensity of anticoagulation to INR: 3–4 (2, 3). In patients with arterial events, another option would be to add low-dose acetylsalicylic acid (LDASA) to the anticoagulant treatment. However, it should be noted that this combination is burdened by a higher bleeding risk (4). Long-term low molecular weight heparin (LMWH) may also be considered as a safe and effective alternative to warfarin, according to the guidelines from the 13th International Congress on Anti-Phospholipid Antibodies (5–9). However, despite anti-thrombotic therapy, 3 to 24% of APS patients still develop recurrent thrombotic events (10–13).

Patients with proven refractoriness to the anticoagulant therapy would benefit from etiological targeted therapies, as aPL are both diagnostic markers and pathogenic drivers of APS (14). Consequently, the 15th International Congress on Anti-Phospholipid Antibodies Task Force on Treatment Trends suggested that immunomodulatory therapy, in addition to or as an alternative to oral anticoagulation, could represent a valuable option for refractory cases (15).

Much of the accumulated experience on the use of immunomodulatory treatments in APS comes from the need to treat refractory patients. Some of them suffer from the catastrophic form of APS but most are carriers of conventional APS with persistent additional symptoms such as migraine, livedo reticularis, and fatigue, which are not included in the latest classification criteria. Most immunomodulatory agents used in APS usually target several steps in B-cell differentiation pathway and their activity. For example, corticosteroids difficult B-cell proliferation and maturation by inhibiting NFkB pathway (16). Other drugs, like hydroxychloroquine, show anti-inflammatory and anti-thrombotic effects (17) by detaching aPL from B2GPI on phospholipid-bound endothelial membranes and protecting annexin V (12). Plasmapheresis removes pathogenic mediators from blood such as autoantibodies and cytokines (18). Intravenous immunoglobulin (IVIG) helps blocking autoantibodies and autoreactive B-cell activation and expansion (19). Among them, hydroxychloroquine is one of the most largely prescribed. It has shown beneficial effects for secondary thromboprophylaxis, especially in SLE patients (20, 21). The combination of glucocorticoids, plasmapheresis or Intravenous immunoglobulins (IVIG) have also shown to be effective in preventing recurrent thrombosis in catastrophic (22) and non-catastrophic APS patients (23). While IVIG is preferred in APS patients with immune thrombocytopenia, plasmapheresis is recommended in microangiopathic hemolytic anemia. Eculizumab, a monoclonal antibody (mAb) blocking complement C5a, has been used for secondary thromboprophylaxis in kidney transplant patients with APS (24). Rituximab, a chimeric mAb targeting CD20 on the surface of B cells, was studied in an open-label phase IIa descriptive pilot study (RITAPS) carried out in 20 patients with non-criteria APS manifestations refractory to conventional treatments (25). The drug proved to be effective in controlling some of symptoms, but levels of aPL remained unchanged (25).

Most of the above-mentioned drugs target B-cell activation, proliferation and/or lower the concentration of circulating antibodies for a short period of time, but they are not directed against the main sources for antibody secretion, which are known to be bone marrow-resident plasma cells (26, 27). These cells express the surface marker CD38 and have the ability to continuously secrete antibodies without further stimulation by their cognate antigen for years or decades. In autoimmunity, as autoreactive B-cells were supposed to be persistently activated by contact with self-antigens, short-lived plasma cells or plasmablasts, which are proliferating cells with a life span of 3 to 5 days (28), were thought to be the most relevant source for autoantibody production. Nonetheless, the clinical experience has proved this hypothesis to be wrong. Treatment with dexamethasone or cyclophosphamide causes a significant reduction in the presence of plasma cells in inflamed tissues, but not in bone marrow niches (27). These untargeted cells continue producing pathogenic autoantibodies that could form immune complexes and reactivate the disease *via* induction of inflammatory pathways in antigen presenting cells or motivate a direct activation of autoreactive memory B-cells (27). Our group previously described the association of thrombosis (29, 30) and non-criteria manifestations (31) in patients with APS and circulating immune complexes with B2GPI.

As the CD38 molecule is expressed on the surface of plasmablasts and plasma cells, although not exclusively (e.g.: on NK cells), they are targeted by daratumumab, an anti-CD38 IgG1-kappa human mAb. The binding of daratumumab to CD38 molecules on the surface of cells raises antibody-dependent cellular cytotoxicity and the depletion of the cells (32). Daratumumab was approved in 2016 in combination with lenalidomide and dexamethasone, or bortezomib and dexamethasone, for the treatment of multiple myeloma patients as a second line therapy (33). However, some off-label uses have been reported in the literature for autoimmune hemolytic anemia (34–37), Evans’ Syndrome (38, 39), cold agglutinin disease (40, 41), pure red cell aplasia (42), immune thrombocytopenia (43), systemic lupus erythematosus (44), and anti-CASPR2 encephalitis (45).

We present the case of a young woman diagnosed of Primary APS with recurrent venous thromboembolic events, in whom the failure of conventional treatment and plasmapheresis led to off-label use of daratumumab.

## Methods

### Patient Consent and Approval of Off-Label Use of Daratumumab

Off-label use of daratumumab was approved by the Medical Direction and the Pharmacy Department of our center. The patient was informed about the goals and possible side effects of the proposed treatment with daratumumab and signed the informed consent. The treatment was funded by our center.

### Blood Samples

Fresh blood samples were drawn from the patient whenever she came to the Hospital and before/after a relevant time point regarding the administered therapy.

### Determination of Anti-Phospholipid Antibodies

The presence of aCL and aB2GPI IgG/IgM was evaluated using a Phadia 250 (ThermoFischer Scientific, MA, USA) with cutoffs established by the manufacturer and validated in our laboratory (99th percentile). LA was measured using HemosIL dRVVT (cutoff ratio 1.2) and HemosIL Silica Clotting Time (cutoff ratio 1.3) assays (Instrumentation Laboratory SpA, Milano, Italy). aPS/PT IgG/IgM antibodies were evaluated using QUANTA Lite ELISA (INOVA DIAGNOSTICS, San Diego, CA, USA) with cutoffs established by the 99th percentile of our local healthy population (IgG: 30 U/mL and IgM 40 U/mL).

### Total Serum Immunoglobulin Quantification

Total serum IgG, IgA, and IgM were evaluated using an Immage 800 nephelometer (Beckman Coulter Fullerton, CA, USA).

### Lymphocyte Subpopulations Study

Lymphocytes were labeled using whole blood (50 µL) and 20 µL of BD multitest 6-color TBNK reagent in Trucount tubes for 15 min. Red blood cells were lysed using fluorescence activated cell sorting lysing solution. Plasmablasts were assessed by a combination of monoclonal antibodies as CD45+CD19+CD38+++IgM− lymphocytes. Determination of lymphocyte subpopulations was performed with a FACSCanto II flow cytometer, and data analyzed by FACSCanto clinical software (BD Biosciences, San Jose, CA, USA).

## Results

### Case Description and Follow-Up

A previously healthy 19-year-old woman taking oral contraceptives was diagnosed of right pulmonary thromoembolism (PTE). As other risk factors such as obesity, smoking or inherited thrombophilia were ruled out, she was investigated for aPL and resulted in positive titers of LA, aCL IgG, aB2GPI IgG and anti-phosphatidylserine/prothrombin (aPS/PT) IgG and IgM. Anti-nuclear antibodies (ANA), complement C3 or C4 consumption or other clinical signs associated to SLE were absent. She was diagnosed of Primary. APS and discharged with acenocoumarol and hydroxychloroquine. Two years later, she developed a new PTE despite adequate INR target. Acenocoumarol was discontinued, and treatment with Low Molecular Weight Heparin (LMWH) and low doses of aspirin were initiated. Four weeks later she was admitted again because of severe left iliofemoral vein thrombosis (VT).

On admission, we switched LMWH to parenteral sodium heparin with well-documented therapeutic anticoagulant activity. On day 1 she began with dyspnea and desaturation. A pulmonary angioCT scan demonstrated the presence of PTE. On day 2, she worsened clinically, and a new angioCT scan demonstrated the growth of the pulmonary thrombus.

To address this worsening clinical picture, we planned a sequential treatment regimen based on two-dose plasmaphereses (days −9 & −8 before the start of treatment with daratumumab) with albumin as reposition, 20 g IVIG (day −4) as a single replacement therapy after plasmapheresis and 4 doses of anti-CD38 daratumumab on days 0, + 7, +14 & +20. The first dose of daratumumab consisted of 8 mg/kg. In the second, third and fourth infusions, 16 mg/kg were administered. The patient experienced a clear improvement of symptoms and signs of VT and PTE from day +1 and could be discharged later on with a therapeutic regimen of subcutaneous enoxaparin (day +8). The overall tolerability of the treatment was adequate. She only developed mild diarrhea with the infusions and one upper respiratory tract infection.

**Figure 1:**
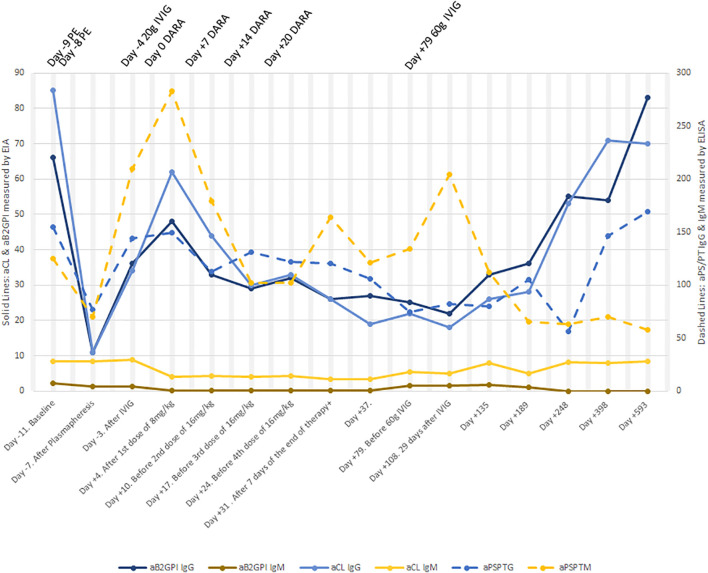
Change in aPL levels before and after therapy. An extreme drop in aPL levels lasting for 4 days was observed after two sessions of plasmapheresis. aPS/PT IgM values even rose to higher levels than in baseline after plasmapheresis. One week after the first dose of daratumumab was administered, aCL IgG and aB2GPI IgG started a decline, this decline lasting until day +108. After day +108, a significant climb began. Levels of aPS/PT IgG lowered until day +248 and then went up again. Levels of aPS/PT IgM showed an intermittent up and down profile, but they have remained stable at half the titer of baseline from day +189 and onwards. PE, plasmapheresis; DARA, daratumumab.

She showed a partial response in her blood analyses from the start of the anti-CD38 therapy with all positive aPL slowly declining. Positive aPL reached their lowest values on day 108 after the first infusion of the drug and 84 days after the end of treatment. Since then, aCL IgG, aB2GPI IgG and aPS/PT IgG began a smooth increase until the time of this report, with aPL values surpassing those from baseline (**Figure 1**). However, aPS/PT IgM values have remained stable on a plateau at half the titer of baseline values.

Almost two years later, and coinciding with the rise of aB2GPI and aCL of IgG isotype, the patient unintentionally missed 6 doses of enoxaparin and was admitted to Hospital with a new VT. Since that episode, she has remained asymptomatic in the follow-ups with an adjusted dose of subcutaneous enoxaparin every 12 hours.

Besides aPL, changes in the total serum immunoglobulins were noted, with a slow decline in total IgG until 4.58 g/L. To avoid susceptibility to infections, the patient was administered a single dose of 60 g polyclonal IVIG on day +79 and has remained within normal levels during the follow-up. Lymphocyte counts did not change significantly but a decline and absence of circulating NK cells was noted after the first infusion. NK count was restored two months after the end of the therapy. As expected by their surface expression of CD38, plasmablasts were removed from peripheral blood and did not show signs of restoration until eight months after the end of therapy. Other B-cell subpopulations did not change significantly (**Table 1**).

**Table 1:** Levels of aPL, serum immunoglobulins, d-dimer and lymphocyte subpopulations.

Time Point	aPS/PT IgG	aPS/PT IgM	aB2GPI IgG	aB2GPI IgM	aCL IgG	aCL IgM	Total Serum IgG	Total Serum IgA	Total Serum IgM	D-Dimer	Total Lymphocyte Count	NK%	NK	CD19%	CD19	Plasma-blasts%
Day −11. Baseline	155	125	66	2.1	85	8.5	8.8	0.9	2.1							
Day −7. After Plasmapheresis	77.1	69.9	11	1.4	11	8.4	3.2	0.5	0.6		**1418**	6.1	87	27.4	389	
Day −3. After IVIG	144	210	36	1.4	34	8.9	10.5	0.8	1	2028						
Day +4. Right after 1st dose of 8 mg/kg	149	283	48	0.1	62	4.1	10.1	1.1	1.5	3497	1631	0.3	5	35.2	575	0.3
Day +10. Before 2nd dose of 16 mg/kg	113	179	33	0.1	44	4.3	7.8	0.5	0.9	878	1656	1.7	28	26.9	446	
Day +17. Before 3rd dose of 16 mg/kg	131	102	29	0.1	30	4.1	6.8	0.3	0.8	397	1728	0.7	12	27.2	470	
Day +24. Before 4th dose of 16 mg/kg	122	102	32	0.1	33	4.3	6.1	0.2	0.6	178	1660	**0.3**	**4**	30	498	
Day +31. After 7 days of the end of therapy	120	164	26	0.1	26	3.4	6.9	0.2	0.8	320	1628	1.2	20	29.1	473	
Day +37.	106	121	27	0.1	19	**3.3**	6.2	0.1	0.7	340	1606	0.8	12	24	386	**0**
Day +79. Before 60 g IVIG	74.2	134	25	1.5	22	5.4	**4.5**	**0.1**	**0.7**	**264**	3116	3.4	108	15.2	475	**0**
Day +108. 29 days after IVIG	82.2	204	**22**	1.6	**18**	5	11.5	0.1	1.1	610	2189	2.6	58	20.1	439	0.1
Day +135	79.6	112	33	1.8	26	8	8.5	0.1	1.1	1280	3069	3.8	117	15.4	475	
Day +189	105	65.5	36	1.1	28	5	6.6	0.2	1.2	201	2500	3.9	98	14.4	362	
Day +248	**56.1**	63.1	55	0.1	53	8.1	6.8	0.3	1.5	563	2003	4.7	95	16	320	0.6
Day +398	146	69.7	54	0.1	71	8	5.9	0.4	1.9	1855	2330	4.4	101	19	442	
Day +593	169	**58.1**	83	0.1	70	8.5	5.7	0.4	1.4	2348	2541	2.9	74	27.7	704	

Bold values represent the lowest levels achieved for every parameter. Besides aPL levels variation, which is described in **Figure 1**, all serum immunoglobulins were affected after treatment with daratumumab, with IgG showcasing the most relevant decline. D-dimer also lowered after treatment and then went up again from day +79 until today. Total lymphocyte count did not change. NK cells and plasmablasts were practically removed from peripheral blood since the first infusion of daratumumab until day +79. aPL are expressed in U/mL. Total serum Immunoglobulins are expressed in g/L and D-dimer in mg/dL. Lymphocyte subpopulations are expressed as % of total lymphocytes or in counts/mL.

## Discussion

As far as we know, this is the first reported use of anti-CD38 therapy in a patient with APS. This treatment was aimed at target plasma cells under the hypothesis that aPL are not only produced by memory B cells but also by bone marrow resident plasma cells. The decision of whether to give rituximab or daratumumab, both off-label uses, for this young lady with repeated PTE and VT despite right anticoagulant therapy, balanced in favor of daratumumab after a careful reading of the evidence. All the studies we found in the literature showed no clear changes in aPL levels after rituximab therapy. Targeting plasma cells also made sense as these cells play an important role in the pathogenesis of autoimmune diseases such as SLE (44, 46), systemic sclerosis (47), Sjögren syndrome (47), ANCA-vasculitis (47), autoimmune cytopenia (34), and rheumatoid arthritis (46).

In our patient, we used a conservative treatment scheme with one dose of 8 mg/kg and three of 16 mg/kg separated by 1 week, with a total duration of the cycle of 1 month. As anti-CD38 mAb therapy was initially developed to kill malignant plasma cells, at the time of administration, we did not have the experience to suggest that a shorter or longer cycle would be effective. At the time of writing of this manuscript, off-label use of daratumumab for autoimmune diseases has increased and some cases have been reported in the literature. In the most recent cases, the authors chose longer cycles with more doses than we proposed for our patient (41).

Clinically, the patient improved from a previously dramatic scenario in which continuous heparin infusion was administered without successful control of the thromboembolic disease.

As a result of the combined interventions made which included two consecutive sessions of plasmapheresis and daratumumab infusions, the patient experienced a remarkable improvement in dyspnea, pleuritic right pain, and the swelling of a leg, so that she could be discharged on day +7 after the start of anti-CD38 therapy. Although we saw an impressive change on the patient’s symptoms right after the first daratumumab infusion, we could not overlook the effect of the combined measures on this initial good outcome.

However, we believe that the beneficial effects observed for two years, with absence of the continuous thrombotic episodes that the patient suffered before the intervention, could be explained by the decrease in aPL titers. Despite we did not achieve a total negativization of aPL, the clinical improvement might be explained by the random depletion of the plasma cell clones producing the more pathogenic antibodies.

On the follow-up, the patient acknowledged incomplete adherence to the treatment with missing of some doses of anticoagulant therapy at multiple time points. One of them led to a new VT two years later, coinciding with the increase in aPL levels, which reached or surpassed baseline levels. This behavior is understandable as we did not target circulating naïve and memory B cells, which would be responsible for the long-term repopulation of aPL. In a recent review on emerging B-cell therapies in SLE by Ayse Bag-Ozbek and Joyce S Hui-Yuen (48), they commented on further treatments employed in two patients with SLE who received four weekly doses of 16 mg/kg daratumumab as part of a clinical trial (44). These two patients were treated with belimumab starting four weeks after the end of therapy with daratumumab to prevent the activation and proliferation of autoreactive B cells.

## Conclusion

The treatment with daratumumab showed an adequate safety profile, was well tolerated, and was associated with clinical improvement, although levels of aPL only showed a partial response. Based on our experience and on data now available in the literature, we suggest that anti-CD38 therapy could be a valuable tool to consider for APS patients who are refractory to the anticoagulant therapy, alone or in combination with B-cell depleting mAbs. Further investigation is needed to better elucidate the role and optimal timing and doses of daratumumab in the treatment of refractory APS.

## Data Availability Statement

The raw data supporting the conclusions of this article will be made available by the authors, without undue reservation.

## Ethics Statement

The studies involving human participants were reviewed and approved by the Hospital Universitario 12 de Octubre, Avenida de Córdoba SN. Madrid, SPAIN. The patient provided her written informed consent to participate in this study.

## Author Contributions

DP and RD-S equally contributed in patient evaluation, therapy administration, follow-up and preparation of this case report. DP and AS designed the strategy of therapy. OC-M, AL, EP-A, CL and AS wrote sections of the manuscript. All authors contributed to the article and approved the submitted version.

## Funding

This study was supported by grant PI17/00147 from “Fondo de Investigaciones Sanitarias” (Institute of Health Carlos III, Spanish Ministry of Economy and Competitiveness), and co-funded with European Regional Development Fund.

## Conflict of Interest

The authors declare that the research was conducted in the absence of any commercial or financial relationships that could be construed as a potential conflict of interest.
